# COVID-19 outbreak investigation and response in a penitentiary setting: the experience of a prison in Italy, February to April 2020

**DOI:** 10.2807/1560-7917.ES.2021.26.38.2001385

**Published:** 2021-09-23

**Authors:** Ruggero Giuliani, Cristina Cairone, Lara Tavoschi, Laura Ciaffi, Teresa Sebastiani, Raffaella Bartolotti, Silvia Mancini, Laura Cremonini, Roberto Ranieri

**Affiliations:** 1 Infectious Diseases Service, Penitentiary Health System, Azienda Socio-Sanitaria Territoriale Santi Paolo e Carlo, Milan, Italy; 2 Prison Health Unit, Azienda Socio-Sanitaria Territoriale Santi Paolo Carlo, Milan, Italy; 3 Department of Translational Research and New Technologies in Medicine and Surgery, University of Pisa, Pisa, Italy; 4 Médecins Sans Frontières Operational Centre Brussels, Brussels, Belgium; 5 Médecins Sans Frontières Italy, Rome, Italy; 6 Welfare General Directorate, Lombardia Regional Health Authority, Milan, Italy

**Keywords:** prison, COVID-19, Italy, outbreak, prevention

## Abstract

Prisons are high-risk settings for COVID-19 and present specific challenges for prevention and control. We describe a COVID-19 outbreak in a large prison in Milan between 20 February and 30 April 2020. We performed a retrospective analysis of routine data collected during the COVID-19 emergency in prison. We analysed the spatial distribution of cases and calculated global and specific attack rates (AR). We assessed prevention and control measures. By 30 April 2020, 57 confirmed COVID-19 cases and 66 clinically probable cases were recorded among a population of 1,480. Global AR was 8.3%. The index case was a custodial officer. Two clusters were detected among custodial staff and healthcare workers. On 31 March, a confirmed case was identified among detained individuals. COVID-19 spread by physical proximity or among subgroups with cultural affinity, resulting in a cluster of 22 confirmed cases. Following index case identification, specific measures were taken including creation of a multidisciplinary task-force, increasing diagnostic capacity, contact tracing and dedicated isolation areas. Expanded use of personal protective equipment, environmental disinfection and health promotion activities were also implemented. Outbreaks of COVID-19 in prison require heightened attention and stringent comprehensive measures.

## Background

Italy was one of the first countries in Europe to be affected by the coronavirus disease (COVID-19) [1] pandemic, with established local virus circulation detected in northern regions in late February 2020 [2]. In particular, Lombardy region, where Milan is located, was the epicentre of the first epidemic wave [2,3].

Early on in the pandemic, attention was drawn to the potential risk of contagion inside prisons, especially when there is overcrowding [4]. Italy ranks third in Europe in prison density, with an occupancy rate of 115% [5-8] and a very old penitentiary infrastructure. Furthermore, the high turnover of people in prison, especially in pre-trial institutions, increases the risk of infectious diseases being introduced [5-7].

COVID-19 has proven to be more severe among older persons and those with comorbidities [2,9]. In Italy, the median age of people in prison is between 50 and 55 years; high rates of acute and chronic physical or mental illnesses and communicable or non-communicable diseases are reported [9-11].

In Italy, where prison healthcare services are managed by the Ministry of Health [12], the custodial system was part of a wider effort to control the COVID-19 epidemic. From the start the Ministry of Justice issued guidelines and procedures to ensure preparedness within the prison system throughout the country, including stringent measures to restrict access to essential staff only and ban visitors [13,14].

### Outbreak detection

In the Lombardy region, with a resident population of ca 10 million, the total prison population numbered 8,720 on 31 January 2020 [5].

Prison A is a pre-trial prison situated in the city of Milan, with a capacity for 840 individuals but an actual population of up to 1,100 people in detention, comprised of men and women taken into custody locally. The average length of detention is around 4 months with 200–300 new incomers admitted per month. The prison infrastructure is old and deficient in several respects, including poor ventilation. The prison consists of six different blocks, each holding 100–200 individuals, divided into sections. People in prison are housed in shared cells (3–8 persons). Custodial staff (CS) are lodged in two separate barracks on prison grounds.

Health services are provided by San Paolo University Hospital (SPH) in Milan and coordinated by the Regional Department of Public Health. Diagnostics for COVID-19, based on PCR on nasopharyngeal swabs, were available throughout the study period for people in prison; testing capacity for prison staff increased during the course of the outbreak.

From 20 February to 30 April 2020, the population in Prison A fell from 1,026 to 764 as a result of the national lockdown (decrease in new arrests, court sittings, new admissions) and of the government’s emergency measures to reduce overcrowding (early release of 5,000 prisoners to home confinement or community supervision) [15]. On 30 March, the prison population numbered 865 (of whom 81 were women), with an average age of 38 years. During the emergency, rehabilitation activities were suspended and detained individuals spent most of their time in their cells. Normally, some 140 people in prison are contracted for in-house catering and general maintenance services and enjoy greater mobility within the prison, including an elevated number of social contacts. We refer to them here as prison workers (PW).

During the COVID-19 emergency, 80 healthcare workers (HCW) rotated to provide essential, urgent and mental healthcare. Among them were two infectious disease specialists and a team of consultants from Médecins Sans Frontières who assisted in developing infection control measures. Custodial staff numbered 535 individuals who covered at least one shift during the study period.

Here we describe the COVID-19 outbreak that occurred in Prison A involving both people in prison and prison staff between 20 February and 30 April 2020 and the bundles of measures taken to contain it.

## Methods

We used the national standard definition of probable and confirmed cases, as updated during the COVID-19 epidemic [16]. Probable cases were all individuals presenting symptoms compatible with COVID-19 and living in, or returning from an area with local transmission of COVID-19, as well as close contacts of confirmed cases. Close contacts in prison settings were defined according to the World Health Organization (WHO) reference document [17]. A confirmed case was defined as an individual (asymptomatic or symptomatic) with a laboratory-confirmed infection with severe acute respiratory syndrome coronavirus 2 (SARS-CoV-2) [17].

As part of COVID-19 prevention and control activities, prison HCW gathered patient data at the time of medical consultation. Data were collected on socio-demographic characteristics (including nationality), virological tests, exposure, symptoms and date of onset, clinical outcomes and movements within the prison of detained individuals and custodial staff (CS) who were reported to be confirmed, probable or possible COVID-19 cases.

Further, we sourced data on registered sick leave requests by custodial staff from the local social security/occupational health database.

Data were entered in Excel and analysed using Stata version 14 (College Station, Texas, United States). We performed a descriptive analysis of the population and the control measures and a retrospective analysis of the temporal distribution of cases by date of diagnosis and date of symptom onset for all cases of COVID-19 identified among people in prison and prison personnel. The attack rate (AR) was calculated as the number of cases per 100 individuals using the Prison A mid-point population (on 30 March) as the denominator. Specific AR were calculated for different populations (CS, HCW and inmates) including probable and confirmed COVID-19 cases.

### Ethical consideration

The study was conducted at the request of regional authorities. This analysis used routine monitoring data collected in collaboration with the local health authorities for the purpose of containing the COVID-19 outbreak. This is standard operating procedure for medical interventions during public health emergencies. Authorisation was obtained from the Ministry of Justice (no. 12107 of 27 February 2020) before data collection. Privacy and confidentiality of patients were ensured. All data were anonymised when entered into the database and identification numbers were coded. No ethnic or other sensitive identifying information was encoded. This retrospective description of programme data is exempt from review by the ethical review board.

## Results

From 20 February to 30 April 2020, a total of 123 COVID-19 cases were identified. Of these, 57 (46.0%) were PCR-confirmed cases among people in prison and CS, and 66 (54.0%) were probable cases among CS who were not tested, owing to suboptimal testing capacity in the early phase of the outbreak, but who had clinically compatible symptoms. The global AR on 30 March was 8.3% (123/1,480) based on the midpoint population (including 865 detainees). The AR was highest among CS with 17.6% (94/535 including probable cases) followed by the HCW with 8.8% (7/80) and people in prison with 2.5% (22/865).

We analysed baseline demographic characteristics of the 123 cases. The median age among the confirmed and probable cases was 44 years (interquartile range: 33–49). All 57 confirmed cases were men, of whom 28 were CS, seven HCW and 22 people in prison. The outbreak affected mainly the age group 40–50 years-old (35.7% of cases: 44/123) followed by the age group 29–39 years-old (34.1%; 42/123).

### Cluster of cases among custodial staff and health workers

**Figure 1:**
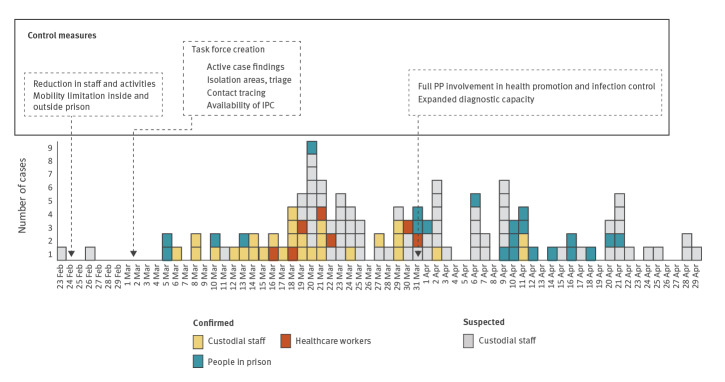
Epidemiological curve in Prison A, Italy, 20 February–30 April 2020 (n = 123)

COVID-19: coronavirus disease; PP: people in prison; PW: prison worker.

The epidemic curve for confirmed and probable cases is shown in Figure 1. The curve has a multimodal shape, suggesting multiple sources of COVID-19 introduction and diverse exposure episodes, each potentially generating clusters of cases.

The x axis shows date of onset or first report of symptoms for symptomatic individuals and date of diagnosis for asymptomatic individuals – mostly people in prison, subject to contact tracing activities.

The first recorded COVID-19 cases were documented among CS at the end of February on the basis of clinical and epidemiological criteria (no access to laboratory confirmation). Clinically ill people were promptly quarantined at home until resolution of symptoms. The first cases (subsequently confirmed) were two members of the unit responsible for the transport of detained individuals to community health services, with onset of symptoms on 23 and 26 of February. Both had probably acquired the infection while performing their duties, while escorting a detained person on a hospital ward and confirmed as a COVID-19 case in hospital on 5 March. More confirmed or probable cases were reported among this unit’s staff, with onset of symptoms after 5 March (Figure 1). Sharing of working spaces (notably vehicles) and, for some, living quarters (barracks), may have been the main route of interpersonal transmission.

A second cluster of cases was identified among CS belonging to a different unit; the first case showed symptoms on 13 March, later confirmed on 25 March. A further 13 probable cases were identified among CS working in the same block. Because of limited testing capacity, only six cases were confirmed. Multiple sources of transmission were identified during the case investigations, including contact with infected family members, possible contact with newcomers to the prison whose status was unknown, sharing of working spaces (e.g. offices, coffee break area) and living quarters (barracks) as well as participation in emergency riot control operations at another prison in Lombardy.

A third cluster of seven confirmed COVID-19 cases was identified among HCW, all with onset of symptoms between 16 and 30 March. The most probable source of transmission was occupational exposure in other healthcare facilities as most HCW were also engaged in community services where infection control measures were poor in the initial phase of the outbreak.

### Cluster of cases among people in prison

**Figure 2:**
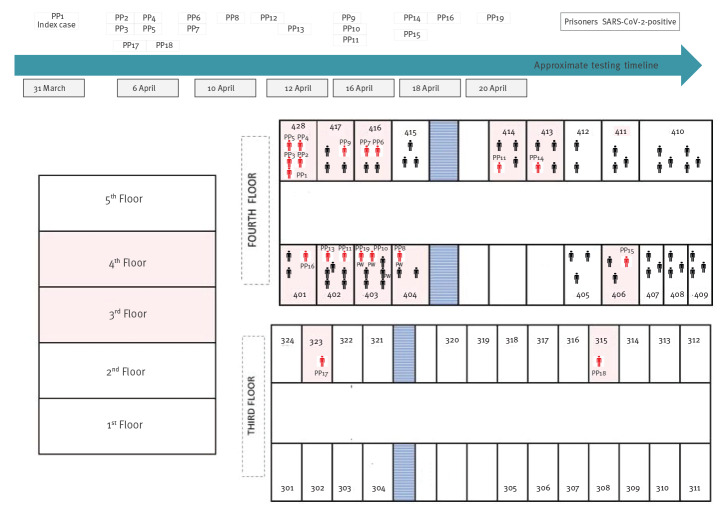
Location of SARS-CoV2 cases within the prison establishment, Milan, 20 February–30 April 2020 (n = 123)

IPC: infection prevention and control; PP: person in prison.

On 31 March, the first probable COVID-19 case in a person in prison (PP1) was identified. This patient was isolated for cough and fever and laboratory-confirmed a few days later. All four of his cellmates in Block X, fourth floor (BX) also complained of mild symptoms and were subsequently confirmed positive (PP2–PP5). Contact tracing activities led to the identification of five more individuals, housed in other cells on the same corridor, as close contacts according to WHO guidance [17], and three tested positive for SARS-CoV-2 (PP6–PP8), one of them being a PW (PP8-PW) (Figure 2). People in prison who socialised with PP8-PW were also identified as close contacts, leading to confirmation of three additional cases (PP9–PP11).

Pink shading: cells and floors inhabited by confirmed COVID-19 cases.

On 11 April, another individual (PP12) housed in BX, fourth floor showed symptoms and tested positive as did an asymptomatic cellmate (PP13). On 18 April, active case finding was expanded to all individuals housed in BX, fourth floor, leading to the identification of three more asymptomatic confirmed cases (PP14–PP16) (Figure 2).

An additional cluster of two cases was identified on the third floor of BX on 6 April (PP17 and PP18). These cases had no link with previous cases, but PP18 reported contact with a symptomatic family member before PP18’s arrest on 11 March, when systematic testing upon admission to the prison had yet to be implemented. The last case of this cluster was an asymptomatic case (PP19) detected after screening all PW, who had recently been transferred from BX fourth floor to the third floor. No further cases were identified among people in prison after 30 April.

Analysis of the spatial distribution of all confirmed COVID-19 cases among people in prison (Figure 2) and of their socio-demographic characteristics suggested that the infection spread by physical proximity (living in the same cell/adjacent cells) or among subgroups with a shared background, including language or geographical origin. All confirmed cases among people in prison involved individuals housed in BX and were concentrated on the fourth (17/19) and third (2/19) floors.

Three additional COVID-19 cases were recorded among the population in Prison A during the study period (Figure 1). However, they were infected while hospitalised at the referral hospital for other clinical reasons.

### Outbreak control measures

After a national emergency was declared at the end of January 2020, preventive measures recommended at national level were implemented in Prison A. In the early phase (29 January–29 February), the number of staff and visitors entering the prison was reduced in order to minimise the risk of introduction of COVID-19. Access to the prison was limited to essential staff (HCW, CS) and all activities not deemed critical were sharply reduced or discontinued. Family visits were initially restricted and subsequently banned and replaced by telephone and Skype calls [14,15]. Referral to community health services was also deferred unless considered urgent while leaves to attend judicial proceedings were suspended and replaced by video calls. Transfers within and between prisons were halted whenever possible both for people in prison and CS.

After the detection of SARS-CoV-2 community circulation in the Lombardy region at the end of February 2020 and the identification of the first COVID-19 cases among prison staff, additional measures were taken (1–30 March), including the creation of a multidisciplinary task force involving both prison and healthcare managers. Protocols were developed to enhance syndromic surveillance among prison staff and people in prison (Table). Active case finding was implemented through the establishment of: (i) an external triage area at the prison to screen all staff upon entry (temperature check and self-assessment questionnaire); (ii) a dedicated triage area for incoming detainees to be tested for COVID-19; (iii) a quarantine area for incoming detainees (14 days quarantine with limited access to communal areas); (iv) a medical isolation area consisting of single cells for probable cases pending virological confirmation; and (v) medical isolation wards for confirmed COVID-19 cases (Table). All prison staff with probable and confirmed COVID-19 were placed on medical surveillance and quarantined. Owing to limited virological testing capacity, re-admission to work was initially based on clearance of clinical symptoms; after 9 April 2021, two successive negative PCR tests were required. Systematic checks were run on all sick leave requests filed by staff, in order to identify probable COVID-19 cases, detect clusters and ensure appropriate re-admission procedures.

**Table:** Bundles of infection control measures adopted in Prison A, Italy, 20 February–30 April 2020 (n = 123)

	Measures	Impact
Preparedness	Create task force including key officers among HCW and CS; identify dedicated areas for triage, quarantine and isolation of COVID-19 confirmed cases; identify most at-risk procedures and dynamics in the prison: develop protocols for active case finding, contact tracing, infection control procedures; staff contingency planning.	Fast decision process and implementation
Limitation of number of possible contacts	Limit movements of people in prison between cells and blocks and access of essential and dedicated staff (CS/HCW) to each block: daily triage for those entering dedicated working area, with symptoms and temperature check; replace family visits and meetings with legal representatives by phone and video calls; restrict staff to certain areas and reduce transfers of people in prison to other cells; movements out of prison allowed only for medical urgency.	Reduce/delay the probability of introducing the virus in the prison
Active case finding	Triage for newly admitted prisoners, with PCR test and isolation for 14 days if PCR test is negative; identify probable cases by syndromic screening and segregate them from their inmates; monitor epidemics among prison staff and ensure contact tracing among prison population and staff.	Rapid identification of cases and prompt isolation
Contact tracing	Identification, isolation and screening of all contacts of confirmed cases among prison population and prison staff.	Contain outbreak spread
Availability of IPC	Ensure supply distribution and proper use of face masks for all prison staff and prisoners; provide alcohol-based hand-rub dispensers on prison premises where appropriate; ensure distance from the cells by using visual signs; develop sanitisation procedure, provide practical training in sanitisation; promote hygiene inside cells and distribute hygiene materials.	Minimise risk for personnel
Communication and coordination	Share information updates with prison staff and people in prison on the state of the epidemic and preparedness plan	Reduce frustration and fear among prison staff and people in prison
Training and education	Train staff in use of PPE, hygiene and preventive measures, environmental sanitisation and cleaning measures, social distancing. Educate people in prison on personal preventive measures (social distancing, hand hygiene, cough etiquette, room cleanliness, use of mask, discouraging exchange of goods and cigarettes).	Reduce risk of transmission

COVID-19: coronavirus disease; CS: custodial staff; HCW: health workers; IPC: infection prevention and control; PCR: polymerase chain reaction; PPE: personal protective equipment.

Infection control procedures were developed focusing on risk evaluation and rational use of personal protective equipment (PPE), initially available in very limited quantities. In-house production of washable masks and coats was undertaken to ensure universal access to PPE. Infection control measures were also introduced for prison staff living in the residential compound (Table).

A rigorous contact tracing procedure was developed in the early stage (28 February 2021) with timely investigation of all confirmed cases, identification and segregation and repeated testing of contact cases (at the latest contact with the index case and after 7 and 14 days) (Table).

The identification of the first COVID-19 case among people in prison at the end of March triggered further measures (31 March–30 April), focused mainly on people in prison working in the communal kitchen and other services but also on the overall prison population. Tailored health promotion messages on infection control were developed and rolled out to explain how and when to use protective masks, increase access to hand hygiene and social distancing and discourage exchanges or sharing of goods among prisoners.

Finally, COVID-19 diagnostic capacity was expanded to increase sampling and testing to rule out asymptomatic infections.

## Discussion

Here we report and describe an outbreak of COVID-19 within a prison institution in Italy and the steps taken to control it. Our work highlights the importance of including prisons in the framework of emergency preparedness [4,18], and reveals some peculiar dynamics of infection transmission in this context.

Closed settings, such as prisons, are at higher risks for the transmission of communicable diseases [19-21], including COVID-19 [22]. At the same time, enforcing control measures such as social distancing, isolation or quarantine, presents logistical and organisational difficulties [23]. In Prison A, detained individuals share common spaces and facilities (e.g. toilets, common showers) both during work and leisure activities. During yard time, physical contacts and exchanges of objects (e.g. cigarettes) occur regularly. Frequent transfers of people in prison to a new section or cell for administrative or disciplinary reasons result in a high rate of intra-prison turnover and increase of potential contacts. Furthermore, certain individuals (PW), are responsible for activities such as shopping or cleaning and enjoy greater freedom of movement on prison grounds with higher risk of acquiring or spreading COVID-19.

The analysis of the chains of transmission among people in prison showed that the infection spread not only among cellmates but also among individuals living in contiguous cells and sharing adjacent spaces. According to our data, transmission was more common between detained individuals with a shared culture or language, forming micro-clusters among people from the same geographical area. The same transmission patterns observed for people in prison, were also detected among CS housed in the prison compound: cases were detected among individuals sharing quarters or having common origins. The complexity of transmission dynamics in prison settings both among people in prison and CS was reported in other COVID-19 outbreaks elsewhere [24].

The CS may have multiple occasions for acquiring SARS-CoV-2 infection outside the prison, including contacts with family and friends. Exposure while performing other duties such as escorting hospitalised individuals was also considered, as nosocomial transmission was predominant especially in the initial stage of the epidemic [25,26]. The temporal distribution of reported cases, with COVID-19 spreading first among CS and then among people in prison, suggests that CS may have introduced the infection into the prison through close contacts during security activities, exchanges of objects or searches in cells, despite social distancing and the use of PPE. In addition, unforeseen critical events, such as the riots in Prison A on 9 March, may have increased the risk of close contacts between CS and between CS and people in prison, thus favouring virus spread at an early stage of the epidemic. It was not possible to identify chains of transmission, however it is likely that asymptomatic/pauci-symptomatic cases among CS and PS may have gone undetected, despite contributing to onward transmission within the prison.

However, as case PP18 suggests, the possibility of initial transmission by a newly admitted asymptomatic individual cannot be ruled out. In fact, COVID-19 probably entered the prison multiple times through different routes but it spread independently among the population of people in prison and the prison staff generating different chains of transmission. The identification and implementation of targeted prevention and control measures was fundamental in responding to the COVID-19 outbreak. We implemented bundles of interventions, in line with international recommendations [17], scaling up the intensity of the response over time. Based on our experience, the presence of a multidisciplinary task force involving both healthcare staff and prison management was essential in controlling the epidemic by ensuring adequate monitoring and risk assessment, coupled with rapid implementation capacity. However, collaboration and engagement should also include people in prison and CS. To avoid misunderstanding and insecurity, all preventive measures should be adequately presented and explained. Educational activities about use of PPE and hygiene measures proved very useful in building awareness of and compliance with infection control procedures and mitigating fear. This is of major relevance considering the additional strain and potential harm associated with prolonged implementation of infection control measures on people already deprived of their liberty. This area has not yet been investigated in depth, but in the aftermath of COVID-19 pandemic it would be appropriate to assess the medium and long-term impact of such measures on the health of people in prison, including mental health [23].

Our findings highlight the importance of active case finding among all population groups, using syndromic surveillance when the supply of diagnostics is limited. Finally, contact tracing was an essential element of the control strategy [27]. In the initial phase of the outbreak, it entailed an individual-based assessment but it was rapidly scaled up to a spatial risk assessment (i.e. covering all detainees in a given block), resulting in increased effectiveness and timely detection of cases.

Our study presents some limitations, mostly deriving from the challenges of operating in a prison setting in an emergency situation. Accuracy and completeness of the data may be suboptimal as many probable cases among correctional staff were never confirmed by PCR but only identified from the social security system. In addition, the transmission chains we found do not follow the order in which the cases were identified, because of the course of asymptomatic presentations and retrospective assessment of symptoms. Confirmatory tests were performed with some delay on account of suboptimal test availability.

## Conclusion

While COVID-19 cases in the prison system may be unavoidable, the challenges of the prison setting require stringent and comprehensive measures, a tailored and multisectoral response involving healthcare prison services and the prison population. The COVID-19 pandemic summons us to fulfil the principle that ‘prison health is public health’ [28] in order to protect the wellbeing of people in prison, staff and the local community, uphold equity and avert the organisational dangers and perils to security and safety that could arise from outbreaks of infectious diseases within the prison environment.
